# Gene2Function: An Integrated Online Resource for Gene Function Discovery

**DOI:** 10.1534/g3.117.043885

**Published:** 2017-06-29

**Authors:** Yanhui Hu, Aram Comjean, Stephanie E. Mohr, The FlyBase Consortium, Norbert Perrimon

**Affiliations:** * *Drosophila* RNAi Screening Center, Department of Genetics, Harvard Medical School, Boston, Massachusetts 02115; † Biological Laboratories, Harvard University, Cambridge, Massachusetts 02138; ‡ Department of Physiology, Development and Neuroscience, University of Cambridge, Cambridge CB2 3DY, United Kingdom; § Department of Biology, Indiana University, Bloomington, Indiana 47405-7005; ** Department of Biology, University of New Mexico, Albuquerque, New Mexico 87131; †† Howard Hughes Medical Institute, Boston, Massachusetts 02115

**Keywords:** functional genomics, orthologs, human genetic disease, model organism databases, data mining

## Abstract

One of the most powerful ways to develop hypotheses regarding the biological functions of conserved genes in a given species, such as humans, is to first look at what is known about their function in another species. Model organism databases and other resources are rich with functional information but difficult to mine. Gene2Function addresses a broad need by integrating information about conserved genes in a single online resource.

The availability of full-genome sequences has uncovered a striking level of conservation among genes from single-celled organisms such as yeast, invertebrates such as flies or nematode worms, and vertebrates such as fish, mice, and humans. This conservation is not limited to amino acid identity or structure, or RNA sequence. Indeed, gene conservation often extends to conservation of biochemical function (*e.g.*, common enzymatic functions); cellular function (*e.g.*, specific role in intracellular signal transduction); and function at the organ, tissue, and whole-organism levels (*e.g.*, control of organ formation, tissue homeostasis, or behavior).

Researchers applying small- or large-scale approaches in any common model organism often come across genes that are poorly characterized in their species of interest. A common and powerful way to develop an hypothesis regarding the function of a gene poorly characterized in one species—or newly implicated in some processes in that species—is to ask whether the gene is conserved and, if so, find out what is known about the functions of its orthologs in other species. This commonly applied approach gains importance when the poorly characterized gene is implicated in a human disease; in many cases, what we know about human gene function is largely based on what was first uncovered for orthologs in other species.

Despite the importance and broad application of this approach among biologists and biomedical researchers, there are barriers to applying the approach to its fullest. First, ortholog mapping is not straightforward. Over the years, many approaches and algorithms have been applied to mapping of orthologs. The results do not always agree and, at a practical level, the use of different genome annotation versions, as well as different gene or protein identifiers, can make it difficult to identify or have confidence in an ortholog relationship. Second, even after one or more orthologs in common model species have been identified, it is not easy to quickly assess in which species the orthologs have been studied and determine what functional information was gained. Model organism databases (MODs) and human gene databases provide relevant, expertly curated information. Although InterMine (Smith *et al.* 2012) provides a mechanism for batch search of standardized information, and NCBI Gene provides information about individual genes in a standardized format, it remains a challenge to navigate, access, and integrate information about all of the orthologs of a given gene in well-studied organisms. As a result, useful information can be missed, contributing to inefficiency and needless delay in reaching the goal of functional annotation of genes, including genes relevant to human disease.

Clearly, there is a need for an integrated resource that facilitates the identification of orthologs and mining of information regarding ortholog function, in particular, in common genetic model organisms supported by MODs. Previously, we developed approaches for integration of various types of gene- or protein-related information, including ortholog predictions [DRSC Integrative Ortholog Prediction Tool (DIOPT); Hu *et al.* 2011], disease–gene mapping based on various sources [DIOPT–Diseases and Traits (DIOPT–DIST); Hu *et al.* 2011], and transcriptomics data [*Drosophila* Gene Expression Tool (DGET); Hu *et al.* 2017]. Importantly, these can serve as individual components of a more comprehensive, integrated resource. Indeed, our DIOPT approach to identification of high-confidence ortholog predictions is now used in other contexts, including at FlyBase (Gramates *et al.* 2017) and at MARRVEL for mining information starting with human gene variant information (Wang *et al.* 2017; www.marrvel.org).

To address the broad need for an integrated resource, we developed Gene2Function (G2F; www.gene2function.org), an online resource that maps orthologs among human genes and common genetic model species supported by MODs, and displays summary information for each ortholog. G2F makes it easy to survey the wealth of information available for orthologs and navigate from one species to another, and connects users to detailed reports and information at individual MODs and other sources. The integration approach and set of information sources are outlined in Figure 1 and Table 1, and described in the Supplemental Material, File S1 (Supplemental Methods).

**Figure 1:**
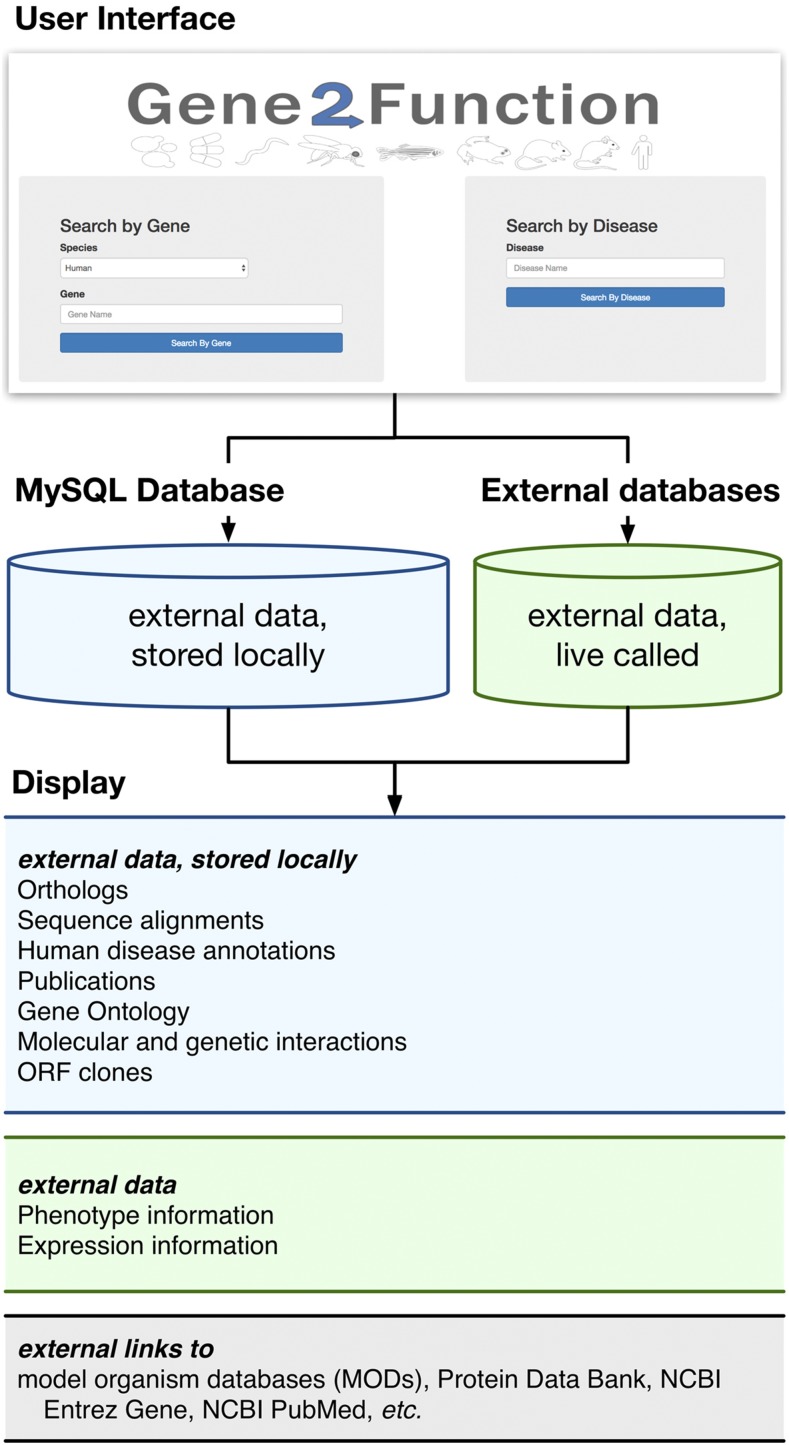
Overview of the Gene2Function (G2F) online resource. For detailed information about the database, logic flow, and information sources, see File S1.

**Table 1:** Summary of disease and gene reports displayed in Gene2Function (G2F)

Column Header	Content	Source of Content
Disease report		
Gene symbol human	Official gene symbol	NCBI gene
Gene ID human	NCBI gene ID	NCBI gene
Count disease terms	Number of disease terms	OMIM, EBI GWAS
Disease terms	Disease terms	OMIM, EBI GWAS
Ortholog overview	Link to G2F gene report	Internal
Gene report		
NCBI gene ID	NCBI gene ID	NCBI gene
Symbol	Official gene symbol	NCBI gene
Human disease counts	Number of disease terms; link to MARRVEL*^a^*	OMIM, EBI GWAS
Species name	Species name	
Species-specific gene ID	Species-specific gene ID	Links to HGNC or MOD gene report*^b^*
Species-specific database	Relevant database name	Links to HGNC or MOD home page
DIOPT score	DIOPT score*^c^*	DIOPT
Best score	Yes or no, this pair has best score at DIOPT	DIOPT
Best score reverse	Yes or no, this pair has best score if opposite search	DIOPT
Confidence	DIOPT confidence*^d^*	DIOPT
Publication count	Number of publications on the ortholog	NCBI gene2pubmed
GO component counts	Number of cellular component GO terms assigned to the ortholog	NCBI gene2go
GO function counts	Number of molecular function GO terms assigned to the ortholog	NCBI gene2go
GO process counts	Number of biological processes GO terms assigned to the ortholog	NCBI gene2go
Protein interaction counts	Number of protein interactions assigned to the ortholog	BioGrid
Genetic interaction counts	Number of genetic interactions assigned to the ortholog	BioGrid
Mine phenotype data	Number of phenotype entries from Mines*^e^*	HumanMine, MouseMine, XenMine, ZebrafishMine, FlyMine, WormBase, SGD
Mine expression data	Number of expression entries from Mines*^e^*	HumanMine, MouseMine, XenMine, ZebrafishMine, FlyMine, WormBase, SGD
Mine disruption phenotype	Number of disruption phenotype entries	UniProt
3D structure	Number of 3D structures available for the ortholog	Protein data bank
ORF clones	Number of ORF clones	PlasmID clone repository*^f^*
Protein alignment	Multiple or pairwise alignment of orthologs	DIOPT

OMIM, Online Mendelian Inheritance in Man; EBI, European Bioinformatics Institute; GWAS, genome-wide association study; HGNC, HUGO Gene Nomenclature Committee; MOD, model organism database; DIOPT, DRSC Integrative Ortholog Prediction Tool; GO, gene ontology; SGD, Saccharomyces Genome Database; ORF, open reading frame.

a MARRVEL, Model organism Aggregated Resources for Rare Variant ExpLoration (Wang *et al.* 2017).

b The databases included at G2F are MGI (Blake *et al.* 2017), RGD (Shimoyama *et al.* 2015), Xenbase (Karpinka *et al.* 2015), ZFIN (Howe *et al.* 2017), FlyBase (Gramates *et al.* 2017), WormBase (Howe *et al.* 2016), SGD (Cherry *et al.* 2012), and PomBase (McDowall *et al.* 2015).

c DIOPT score, number of ortholog prediction tools included at DIOPT (Hu *et al.* 2011) that cover both species and predict the displayed ortholog match.

d In this column, “High” indicates that the ortholog pair has the best score among all pairs with both a forward and a reverse direction score and a DIOPT ≥ 2; “Moderate” indicates that the ortholog pair has the best score with the forward or the reverse search and a DIOPT ≥ 2, or has a DIOPT score ≥ 4 but is not the best score with either a forward or reverse search; and “Low” includes all other predicted ortholog pairs.

e Mines (or MODs serving that function): HumanMine, MouseMine, XenMine, ZebrafishMine, FlyMine, WormBase, and SGD (Cherry *et al.* 2012; Smith *et al.* 2012; Howe *et al.* 2016).

f Links provided for one of several repositories in the United States and overseas that have ORF clones, many of which are from the ORFeome Collaboration (2016).

To demonstrate the utility of G2F, we focus on two use cases: (1) a search initiated with a single human or common model organism gene of interest, and (2) a search initiated with a single human disease term of interest.

A gene search at G2F connects users to ortholog information and an overview of functional information for orthologs (Table 1). Specifically, starting with a search of a human, mouse, frog, fish, fly, worm, or yeast gene, users reach a summary table of orthologs and information. Information displayed includes the number of gene ontology (GO) terms assigned based on experimental evidence; the number of publications; and the number of molecular and genetic interactions reported. When available, the table also includes links to expression pattern annotations, phenotype annotations, three-dimensional structure information (Rose *et al.* 2017), and open reading frame (ORF) clones from the ORFeome collaboration consortium (Lamesch *et al.* 2004; Hu *et al.* 2007; ORFeome Collaboration 2016) which are available in a public repository (Zuo *et al.* 2007). The summary allows a user to quickly (1) evaluate conservation across major model organisms based on DIOPT score, pairwise alignment of the query protein to another species, and multiple-sequence alignment; (2) assess in what species the query gene has been well studied based on original publications, annotation, and data; and (3) identify reagents for follow-up studies. The summary table also allows a user to view detailed reports and is hyperlinked to more detailed information at original sources, such as data on specific gene pages at MODs.

A disease search at G2F first connects from disease terms to associated human genes, then uses the gene search results table format to display orthologs of the human gene and summary information (Table 1). After a search with a human disease term, users are first shown a page that helps to disambiguate terms, expanding or focusing the search, and also allows users to limit the results to disease–gene relationships curated in the Online Mendelian Inheritance in Man database and/or based on genome-wide association studies (GWAS) from the National Human Genome Research Institute–European Bioinformatics Institute GWAS Catalog (MacArthur *et al.* 2017). Next, users access a table of human genes that match the subset of terms, along with summary information regarding the genes and associated disease terms. On the far right-hand side of the table, users can connect to the same single gene-level report that is described above for a gene search.

Over the past two decades, GWAS have begun to reveal genetic risk factors for many common disorders (Wangler *et al.* 2017). As of February 2017, the GWAS Catalog (MacArthur *et al.* 2017) included 2385 publications, with 10,499 reported genes associated with 1682 diseases or traits. For some of the human genes, there are no publications or GO annotations. We used G2F to survey information in model organisms for this subset of genes and found many cases where one or more orthologous genes have been studied (File S1). The results of the ortholog studies appear in some cases to support the disease association, and the corresponding model systems could provide a foundation for follow-up studies (Table S1). The human gene *SAMD10*, for example, has been shown (using the iCOGS custom genotyping array) to be one of 23 new prostate cancer susceptibility loci (Eeles *et al.* 2013), but there is no information about this human gene available, aside from sequence and genome location. The results of a G2F search show that the gene is conserved in the mouse, rat, fish, fly, and worm. The mutant phenotypes of the fly ortholog suggest that the gene is involved in compound eye photoreceptor cell differentiation, EGFR signaling, positive regulation of Ras signaling, and ERK signaling, providing starting points for the development of new hypotheses regarding the function of *SAMD10*. Several uncharacterized human genes associated by GWAS with schizophrenia, namely *IGSF9B*, *NT5DC2*, *C2orf69*, and *ASPHD1* (Ripke *et al.* 2013; Schizophrenia Working Group of the Psychiatric Genomics Consortium 2014), are expressed at higher levels in the nervous system than in other tissues in one or more model organisms, suggesting a potential role in the nervous system in these models and supporting the idea that the models might be appropriate for follow-up studies aimed at understanding human gene function. These examples are extreme in that they represent human genes for which there are no publications describing functional information. For a large number of human genes, limited information is available. Functional annotations in model systems, as accessed through G2F, can help in the development of new hypotheses regarding the functions of these genes, as well as help researchers to choose an appropriate model organism or organisms for further study of the conserved gene.

Altogether, G2F provides a highly integrated resource that facilitates efficient use of existing gene function information by providing a big-picture view of the information landscape and building bridges between different islands of information, including MODs. This approach complements approaches designed for searches starting with long gene lists (*e.g.*, InterMine; Smith *et al.* 2012) or those based on a phenotype-centered model (*e.g.*, the Monarch Initiative; Mungall *et al.* 2017). The modular nature of the G2F resource makes it possible to easily update the information sources (*e.g.*, replace a module) and add new types of information (*e.g.*, an expanded summary of reagents or new types of experimental data).

## Supplementary Material

Supplemental material is available online at www.g3journal.org/lookup/suppl/doi:10.1534/g3.117.043885/-/DC1.
