# Supplementary material for: Gene2Function: An Integrated Online Resource for Gene Function Discovery
Source: G3 (Bethesda). 2017 Jun 29;7(8):2855–8. doi: 10.1534/g3.117.043885 (PMC5555488; doi:10.1534/g3.117.043885)
Supplement: Supplementary file 1 [file 2855FileS1.docx]

**Supplemental Methods**

***Structure and logic***

Gene2Function (G2F) was built using php, python and javascript with a backend MySQL database. Jquery and Silex framework libraries were used for software development. To respond to the Ajax requests for mine data, a Python script was developed with the InterMine webservice package to query data from the various InterMine sources. The G2F site is hosted by the Harvard Medical School Research Computing group. See also ***Fig. 1*** and ***Table 1*** in the main text. The core coding components and documentation of the data structure, as well as instructions for setting up the database, are available on github ([https://github.com/DRSC-FG/gene2function](https://github.com/DRSC-FG/gene2function)).

***Information sources***

**Orthologs.** Ortholog mapping is based on the DRSC Integrative Ortholog Prediction Tool (DIOPT), originally published in 2011 (Hu *et al.* 2011) and regularly updated to cover more organisms, to integrate more ortholog prediction algorithms, and to synchronize with new genome annotations (Hu *et al.* 2017). Currently, DIOPT integrates 14 different ortholog prediction algorithms for 9 major model organisms and calculate a simple voting score to reflect the strength of orthologous relationships. Gene2Function display the orthologous relationships supported by at least 3 algorithms and only display weak associations (score 1 or 2) when there is no better option. Besides the voting score, DIOPT also provides pairwise and multiple sequence alignments for G2F.

**Human disease-gene associations.** Mapping of human diseases to associated genes is done based on our DIOPT-Diseases and Traits (DIOPT-DIST) approach (Hu *et al.* 2011). Two types of disease-gene associations are included in the results; first, gene-disease associations annotated at OMIM (<https://omim.org/downloads/)>, and second, reported gene-disease or gene-trait associations from the catalog of genome-wide association studies (GWAS Catalog, https://www.ebi.ac.uk/gwas/docs/file-downloads).

**Detailed gene information pages.** G2F provides links to detailed, expert-curated information pages for individual genes in human or model organism databases (MODs). The following sources were used: for human genes, the Human Gene Nomenclature Committee (HGCN) resource (genenames.org/) (Yates *et al.* 2017); for mouse genes, the Mouse Genome Database (MGD) resource (informatics.jax.org/) (Blake *et al.* 2017); rat, the Rat Genome Database (RGD) (rgd.mcw.edu/) (Shimoyama *et al.* 2015); frog, Xenbase (xenbase.org) (Karpinka *et al.* 2015); zebrafish, ZFIN (zfin.org) (Howe *et al.* 2017); for *Drosophila*, FlyBase (flybase.org) (Gramates *et al.* 2017); for *C. elegans*, WormBase (wormbase.org) (Howe *et al.* 2016); for *S. cerevisiae*, the Saccharomyces Genome Database (SGD) (yeastgenome.org/) (Cherry *et al.* 2012); for *S. pombe*, PomBase (pombase.org) (McDowall *et al.* 2015).

**Gene ontology terms.** Gene ontology (GO) terms were downloaded from NCBI ftp site (ftp://ftp.ncbi.nlm.nih.gov/gene/DATA/gene2go.gz). A java program was developed to parse the file, select the records relevant to the 9 species covered by G2F, and format the file for database upload. A script is scheduled to run monthly to automatically update the database. Only gene ontology assignments based on experimental evidence are selected and displayed at G2F; these include assignments based on the evidence codes EXP, IDA, IPI, IMP, IGI and IEP.

**PubMed publications.** Gene-associated publications are retrieved from NCBI ftp site (<ftp://ftp.ncbi.nlm.nih.gov/gene/DATA/gene2pubmed.gz>). A java program was developed to parse the file, select the records relevant to the 9 species covered by G2F, and format the file for database upload. A script is scheduled to run monthly to automatically update the database. At the user-interface, the Pubmed IDs are sorted in descending order so that more recent publications appear at the top. We filtered out publications with >100 associated genes so that the publication counts reflect the type of low- to medium-throughput studies most likely to include functional characterization.

**Protein and genetic interaction annotations.** Protein-protein interaction and genetic interaction annotations are from BioGrid (<https://thebiogrid.org/)> (Chatr-Aryamontri *et al.* 2017).

**Phenotype and expression annotations.** Phenotype and expression annotations for human, mouse, zebrafish, and *Drosophila* genes are retrieved using application program interfaces (APIs) provided by InterMine (<http://intermine.org/)> (Smith *et al.* 2012). The phenotype and expression annotations for worm and budding yeast genes are provided by direct link out to MODs (Cherry *et al.* 2012; Howe *et al.* 2016), whereas expression data for fission yeast genes are retrieved from PomBase (McDowall *et al.* 2015) and stored locally.

**Disruption phenotype and 3D structure annotation.** Disruption phenotype and 3D structure annotation are queried from UniProt website directly (http://www.uniprot.org/) (UniProt Consortium 2017). The information were processed, formatted and stored locally, which is subjected to periodically update.

**Reagent information.** Open reading frame (ORF) clone information was obtained from the Dana Farber/Harvard Cancer Center DNA Resource Core PlasmID database (https://plasmid.med.harvard.edu/PLASMID/) (Zuo *et al.* 2007). We include in the G2F report only ORFs in gateway vectors, majority of which were from the ORFeome collaboration (OC) consortium (Collaboration 2016). In addition to genome-scale human ORF clones, large-scale collections for several other model organisms (mouse, *Drosophila*, and the yeast *S. cerevisiae*) are also included in G2F.

***GWAS gene function analysis***

To generate ***Table S1***, we first retrieved genes from the NHGRI-EBI GWAS Catalog (Feb 27^th^ 2017) (MacArthur *et al.* 2017) that we have stored locally at DIOPT-DIST (Hu *et al.* 2011). Genes associated with traits that are not categorized as diseases, such as normal phenotypes, disease risk factors, diagnosis, and treatment, were filtered out. For the remaining 6131 genes, we surveyed publication counts and GO annotations. Based on this, we classified 293 of the 6131 genes as ‘unstudied’ based on the fact that they had no associated qualified publications (see above for publication selection criteria) and no experimental-based gene ontology annotations. 58 of the 293 genes can be mapped to model organism(s) with high or modest DIOPT rank (<http://www.flyrnai.org/DRSC-ORH.html#versions>). We looked at information available for these orthologous genes using G2F and used this to select the 12 highly conserved candidates included in ***Table S1***.

**Reference Citations for Supplemental Methods**

Blake, J. A., J. T. Eppig, J. A. Kadin, J. E. Richardson, C. L. Smith *et al.*, 2017 Mouse Genome Database (MGD)-2017: community knowledge resource for the laboratory mouse. Nucleic Acids Res 45**:** D723-D729.

Chatr-Aryamontri, A., R. Oughtred, L. Boucher, J. Rust, C. Chang *et al.*, 2017 The BioGRID interaction database: 2017 update. Nucleic Acids Res 45**:** D369-D379.

Cherry, J. M., E. L. Hong, C. Amundsen, R. Balakrishnan, G. Binkley *et al.*, 2012 Saccharomyces Genome Database: the genomics resource of budding yeast. Nucleic Acids Res 40**:** D700-705.

Collaboration, O. R., 2016 The ORFeome Collaboration: a genome-scale human ORF-clone resource. Nat Methods 13**:** 191-192.

Gramates, L. S., S. J. Marygold, G. D. Santos, J. M. Urbano, G. Antonazzo *et al.*, 2017 FlyBase at 25: looking to the future. Nucleic Acids Res 45**:** D663-D671.

Howe, D. G., Y. M. Bradford, A. Eagle, D. Fashena, K. Frazer *et al.*, 2017 The Zebrafish Model Organism Database: new support for human disease models, mutation details, gene expression phenotypes and searching. Nucleic Acids Res 45**:** D758-D768.

Howe, K. L., B. J. Bolt, S. Cain, J. Chan, W. J. Chen *et al.*, 2016 WormBase 2016: expanding to enable helminth genomic research. Nucleic Acids Res 44**:** D774-780.

Hu, Y., A. Comjean, C. Roesel, A. Vinayagam, I. Flockhart *et al.*, 2017 FlyRNAi.org-the database of the Drosophila RNAi screening center and transgenic RNAi project: 2017 update. Nucleic Acids Res 45**:** D672-D678.

Hu, Y., I. Flockhart, A. Vinayagam, C. Bergwitz, B. Berger *et al.*, 2011 An integrative approach to ortholog prediction for disease-focused and other functional studies. BMC Bioinformatics 12**:** 357.

Karpinka, J. B., J. D. Fortriede, K. A. Burns, C. James-Zorn, V. G. Ponferrada *et al.*, 2015 Xenbase, the Xenopus model organism database; new virtualized system, data types and genomes. Nucleic Acids Res 43**:** D756-763.

MacArthur, J., E. Bowler, M. Cerezo, L. Gil, P. Hall *et al.*, 2017 The new NHGRI-EBI Catalog of published genome-wide association studies (GWAS Catalog). Nucleic Acids Res 45**:** D896-D901.

McDowall, M. D., M. A. Harris, A. Lock, K. Rutherford, D. M. Staines *et al.*, 2015 PomBase 2015: updates to the fission yeast database. Nucleic Acids Res 43**:** D656-661.

Shimoyama, M., J. De Pons, G. T. Hayman, S. J. Laulederkind, W. Liu *et al.*, 2015 The Rat Genome Database 2015: genomic, phenotypic and environmental variations and disease. Nucleic Acids Res 43**:** D743-750.

Smith, R. N., J. Aleksic, D. Butano, A. Carr, S. Contrino *et al.*, 2012 InterMine: a flexible data warehouse system for the integration and analysis of heterogeneous biological data. Bioinformatics 28**:** 3163-3165.

UniProt Consortium, T., 2017 UniProt: the universal protein knowledgebase. Nucleic Acids Research 45**:** D158-D169.

Yates, B., B. Braschi, K. A. Gray, R. L. Seal, S. Tweedie *et al.*, 2017 Genenames.org: the HGNC and VGNC resources in 2017. Nucleic Acids Res 45**:** D619-D625.

Zuo, D., S. E. Mohr, Y. Hu, E. Taycher, A. Rolfs *et al.*, 2007 PlasmID: a centralized repository for plasmid clone information and distribution. Nucleic Acids Res 35**:** D680-684.
